# Advanced Molecular Knowledge of Therapeutic Drugs and Natural Products Focusing on Inflammatory Cytokines in Asthma

**DOI:** 10.3390/cells8070685

**Published:** 2019-07-05

**Authors:** Sheng-Chieh Lin, Li-Shian Shi, Yi-Ling Ye

**Affiliations:** 1 Division of Allergy, Asthma and Immunology, Department of Pediatrics, Shuang Ho Hospital, Taipei Medical University, Taipei 23561, Taiwan; 2 Department of Pediatrics, School of Medicine, College of Medicine, Taipei Medical University, Taipei 11031, Taiwan; 3 Graduate Institute of Clinical Medicine, College of Medicine, National Taiwan University, Taipei 10002, Taiwan; 4 Department of Biotechnology, National Formosa University, Yunlin 63201, Taiwan

**Keywords:** asthma, cytokines, antibodies, interleukin, thymic stromal lymphopoietin, herbs, natural compounds

## Abstract

Asthma is a common respiratory disease worldwide. Cytokines play a crucial role in the immune system and the inflammatory response to asthma. Abnormal cytokine expression may lead to the development of asthma, which may contribute to pathologies of this disease. As cytokines exhibit pleiotropy and redundancy characteristics, we summarized them according to their biologic activity in asthma development. We classified cytokines in three stages as follows: Group 1 cytokines for the epithelial environment stage, Group 2 cytokines for the Th2 polarization stage, and Group 3 cytokines for the tissue damage stage. The recent cytokine-targeting therapy for clinical use (anti-cytokine antibody/anti-cytokine receptor antibody) and traditional medicinal herbs (pure compounds, single herb, or natural formula) have been discussed in this review. Studies of the Group 2 anti-cytokine/anti-cytokine receptor therapies are more prominent than the studies of the other two groups. Anti-cytokine antibodies/anti-cytokine receptor antibodies for clinical use can be applied for patients who did not respond to standard treatments. For traditional medicinal herbs, anti-asthmatic bioactive compounds derived from medicinal herbs can be divided into five classes: alkaloids, flavonoids, glycosides, polyphenols, and terpenoids. However, the exact pathways targeted by these natural compounds need to be clarified. Using relevant knowledge to develop more comprehensive strategies may provide appropriate treatment for patients with asthma in the future.

## 1. Introduction to Asthma

Asthma is a common respiratory disease worldwide. It clinically manifests as wheezing, nocturnal cough, shortness of breath, chest tightness, and variable expiratory airflow limitation [1]. An estimated 300 million people have asthma worldwide [2,3]. In the Unites States, the average annual prevalence of asthma is approximately 9.5% in children and approximately 7.7% in adults [4]. Asthma is the most common chronic disease among children. The prevalence of asthma in children showed marked geographic variation from 2% to 32% in different countries [5]. In 2008, an average of four missed school days for children and five missed work days for adults because of asthma were noted in the United States [6,7], which accounts for 10.5 million missed school days and 14.2 million missed workdays due to asthma attacks [6,7]. It is essential to control asthma, but poverty, inadequate health care, culture, and environmental pollutants are barriers to reducing the burden of asthma [5]. Significant progress has been made in understanding asthma. However, the cause of the disease is still unclear, and more information is needed regarding the complex interrelationship of the immunologic, genetic, environmental, and pharmacologic factors involved in the disease [8]. 

### 1.1. The Molecular and Cellular Basis for Asthma

**Figure 1:**
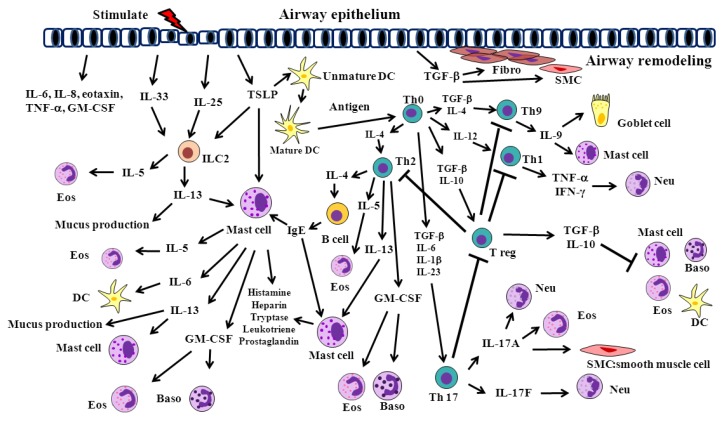
Cytokine profiles in the development of asthma.

Asthma is characterized by allergy, airway hyperreactivity, inflammation, remodeling of the airways of the bronchus, and the number of immune cells increased in the airway [9]. Many immune cells including dendritic cells (DCs), T cells, B cells, eosinophils, basophils, neutrophils, and mast cells infiltrate the submucosa of the bronchus and cause a series of immune reactions in asthma [9,10]. The presence of inflammatory cells in the airway results in an altered repair response, with the secretion of cytokines and growth factors that induce varying structural changes to the airways, which is termed as airway remodeling [11,12]. Airway remodeling includes elevated numbers of inflammatory cells, a hypertrophy of submucosal glands, goblet cell hyperplasia, hyperplasia of the airway smooth muscle, and the deposition of collagen and fibronectin in the subepithelial basement membrane or in the submucosa around or within airway smooth muscle bundles [9,11,13,14,15]. Abnormal extracellular matrix component depositions have crucial roles in the thickness of the airway smooth muscle [16]. The pathogenesis of asthma includes pathways of innate immunity, adaptive immunity, and memory immunity. Initially, the airway of a fetus does not contain DCs. After birth, microbes and irritants activate the respiratory epithelium. The main innate immunologic stimuli initiate the ingression of immature DCs from the bone marrow [17,18]. When airway tissues are damaged or undergo cellular stress or cell death, respiratory epithelial cells (ECs) express many pattern recognition receptors to rapidly detect and respond to pathogen-associated molecular patterns or damage-associated molecular patterns [19]. The overexpression of epithelial pattern recognition receptors leads to the secretion of cytokines, chemokines, and antimicrobial peptides [19]. Chemoattractants such as chemokine (C–C motif) ligand 19, 20, and 27 (CCL19, CCL20, and CCL-27, respectively) and the ligands for C–C chemokine receptor type 6, 7, and 10 (CCR6, CCR7, and CCR10, respectively) direct DCs migration toward the damaged epithelium and underlying mucosa [17,18]. In response to the damage, airway ECs release the cytokines of interleukin (IL)-6, IL-8, granulocyte-macrophage colony-stimulating factor (GM-CSF), eotaxin, and tumor necrosis factor-α (TNF-α) to activate and recruit more immune cells [20]. IL-6 is crucial for DCs to trap allergens and initiate Th2/Th17-mediated airway inflammation and airway hyperresponsiveness (AHR) in asthma [21]. Cytokines such as thymic stromal lymphopoietin (TSLP), IL-25, and IL-33 from damaged ECs stimulate myeloid DC maturation and activation [17]. TSLP, IL-25, and IL-33, which are all EC-derived cytokines and chemokines, play crucial roles in the initiation of innate immunity. In the human lung, IL-33 is predominantly expressed by bronchial ECs [10]. IL-33 is considered a damage-associated molecular pattern, and it has two major domains—an IL-1-like domain and an N-terminal nuclear domain—that activate the immune response after cellular injury [22]. After allergen exposure, IL-33 is rapidly released into the airway within one hour, which involves an increase in its concentration in the bronchoalveolar lavage fluid [22]. IL-33 may stimulate Group 2 innate lymphoid cells (ILC2) to increase the production of Th2-type cytokines, such as IL-13 and IL-5, in the innate allergic immune response [10]. IL-25 is a member of the IL-17 family and is similar to IL-33; it is expressed by airway ECs [23]. IL-25 is released when the cell is exposed to protease-containing antigens, such as house dust mite, and it is released by immune cells such as Th2 cells, mast cells, basophiles, and eosinophils [23,24]. TSLP is a four-helix bundle cytokine that can activate DCs, NKT cells, mast cells, and eosinophils to interact with cytokines and inflammatory mediators on the airway smooth muscle of patients with asthma [25]. In an asthma model, TSLP can upregulate natural killer T cells to increase IL-13 production and decrease airway hyperreactivity [26]. TSLP directly activates mast cells and induces mast cells to release multiple proinflammatory cytokines and chemokines independent of immunoglobulin E (IgE) [27]. TSLP can stimulate human eosinophils through the nuclear factor-κB (NF-κB)-dependent signaling pathway and the activation of extracellular signal-regulated protein kinase, namely p38 mitogen-activated protein kinase [26]. TSLP-like IL-33 and IL-25 can also stimulate ILC2 to increase the production of Th2-type cytokines [16]. In patients with asthma, re-exposure to allergens (such as dust mites, animal dander, dust, mold spores, and pollen) or environmental stimuli can cause an adaptive immune response [25]. Environmental antigens are immune-regulated, and are recognized by DCs, macrophages, B lymphocytes, and several other cell types that belonged to antigen presenting cells (APCs) [17]. APCs present antigens to CD4 positive (CD4+)Th2 cells through the endocytic pathway by processing eight to 10 amino acid epitopes in major histocompatibility complex (MHC) class II molecules. In allergic asthmatic patients, DCs play a crucial role in activating naive T cells. TSLP-activated DCs show stronger potential to express the OX40 ligand (OX40L; CD252) and can trigger the differentiation of naive CD4+ T cells into inflammatory CD4+ T helper type 2 (Th2) cells and the expansion of allergen-specific Th2 memory cells [28]. After this processing, Th2 cells-like mast cells and basophils produce IL-4, inducing B lymphocytes to switch antibody production and resulting in immunoglobulin class switching from IgM to IgE [29]. IgE binds to high-affinity and low-affinity receptors on mast cells, basophils, and eosinophils, causing calcium influx and degranulation. These cells release inflammatory mediators such as histamine, heparin, tryptase, prostaglandin, and leukotriene, which induce airway smooth muscle contraction, vasodilatation, mucus secretion, and increased vascular permeability. Th2 cells release various proallergic inflammatory cytokines, such as IL-4, IL-5, IL-13, and GM-CSF, which activate basophils and eosinophils and increase mucus secretion in the airway in patients with asthma [30]. IL-4 not only induces IgE production, but also stimulates the differentiation of naive T cells into Th2 cells and initiates the expression of vascular cell adhesion protein 1 (VCAM-1) to direct the migration of T cells, monocytes, basophils, and eosinophils to allergic inflammation sites [31]. IL-5 can stimulate eosinophil production and contribute to the differentiation, proliferation, and survival of eosinophils [32]. IL-13 has functions similar to those of IL-4, and can increase AHR [30]. Naive T cells can also differentiate into Th9 cells, Th17 cells, and regulatory T cells (Treg) cells. Th9 cells secrete IL-9 to stimulate mucus production, goblet cell hyperplasia, and mast cell development [33]. Th17 cells secrete IL-17A to stimulate eosinophils, and these cells influence airway smooth muscle [17]. Th17 cells overexpress IL-17A and IL-17F, which may aggravate the neutrophil inflammatory response [34]. Th1 cells secrete TNF-α and interferon –γ (IFN-γ) to activate neutrophils [17]. Treg cells can inhibit the functions of TH1, Th17, and Th9 cells, and can secrete IL-10 and transforming growth factor-beta (TGF-β) to inhibit Th2 cells [17] After the series of immune reactions, some lymphocytes become memory T or B cells, which show immediate responses to the next allergen exposure. Sensitized ECs also release the fibroblast growth factor endothelin and TGF-β, which lead to the release of proteoglycans, glycoproteins, and collagen that cause airway remodeling [35]. A disintegrin and metalloproteinase-33 (ADAM-33), TGF-β, vascular endothelial growth factor, matrix metalloproteinase-9 (MMP-9), IL-5, IL-13, and IL-14 are key mediators involved in airway remodeling in asthma [35]. In a recent study, unregulated levels of connective tissue growth factor (CTGF) correlated with the MMP-9 level were found in the airway remodeling of asthma (Figure 1) [36]. 

Asthma is believed to be a chronic disease caused by the separate responses of innate and adaptive immunity to allergens; however, this concept has changed based on reports of ILC2 [37,38]. ILC2 are innate cells that can produce allergic cytokines without the need of adaptive T cell and B cell products [37,38]. TSLP activates DCs through TSLPR and promotes DCs to cause the differentiation of naive CD4+ T cells into TH2 cells to secrete Th2 cytokines, promoting the selective expansion of TH2 cells [39]. TSLP, IL-25, and IL-33 can directly stimulate ILC2 to secrete Th2 cytokines, and they induce antigen-specific IL-5 CD4+ T cells and promote allergen-induced inflammation independent of IL-4 [40]. TSLP may play a fundamental role in the innate–adaptive interface in the pathology of asthma [25]. 

### 1.2. Signaling Pathways Involved in Cytokine Activity during Asthma Development

Although many signaling pathways are involved in the development of asthma, we proposed three major signaling pathways for these three stages of asthma that we mentioned above. TSLP production by monocyte-derived DCs requires the integration of signals from dectin-1, the IL-1 receptor, and ER stress signaling pathways [41]. The NF-κB pathway is the first pathway that involves many epithelium-stage proinflammatory cytokines. NF-κB signaling is the first pathway in chronic inflammatory airway disease [42]. Also, the activation of DCs requires induction of the pro-inflammatory transcription factor NF-κB [43]. In both asthma and chronic obstructive pulmonary disease, oxidative stress contributes to airway inflammation by inducing inflammatory gene expression. NF-κB is an essential participant involved in many inflammatory networks involving chemokines (e.g., IL-8, macophage inflammatory protein 1 alpha (MIP-1α), monocyte chemoattractant protein 1 (MCP1), regulated on activation, normal T cell expressed and secreted (RANTES), and eotaxin), pro-inflammatory cytokines (e.g., IL-1, IL-2, IL-6, and TNF-α), adhesion molecules (e.g., intercellular adhesion molecule (ICAM), VCAM), and E-selectin), and inducible pro-inflammatory enzymes (COX-2 and iNOS), which regulate cytokine activity in airway inflammation [44]. The T cell development during the second and third stages are endotype-dependent [45]. 

The second signaling pathway is the GATA3 and janus kinase/signal transducers and activators of transcription (JAK–STAT) pathways. Atopic asthma is associated with high levels of Th2 cells. GATA3 controls cellular function and predominantly promotes Th2 differentiation [46]. In the study of Shrine et al., the identification of the GATA3 and KIAA1109 signals are associated with moderate-to-severe disease [47]. Th2 cell activation occurs through JAK–STAT signaling. Targeting this pathway through the inhibition of cytokines (IL-4 and IL-13) and their receptors, JAKs or STATs, has been shown to have a therapeutic effect on asthma pathology [48]. The major JAK–STAT signaling pathway involved in the asthmatic response is the IL-4/IL-13/STAT6 pathway [49]. Several studies have explored the origins of Th17 cells in severe asthma. IL-1β and IL-6, each of which are critical to Th17 differentiation, are expressed at high levels in the inflamed airways of children with severe asthma [50]. Transcriptional factors such as RAR-related orphan receptor gamma (RORγt), STAT3, RAR-related orphan receptor alpha (RORα), and Interferon regulatory factor 4 (IRF-4), -are all involved in Th17 differentiation.

The final stage is correlated with Smad2/3-related signaling involving TGF-β. Also, the role of regulatory T cells (Tr) in this stage is also important.^45^ TGF-β regulates multiple cellular processes such as EC growth suppression, alveolar ECs differentiation, fibroblast activation, and extracellular matrix organization that is closely associated with tissue remodeling in pulmonary fibrosis and emphysema [51]. The polymorphism of Smad3, which is involved in TGF-β signaling, is associated with asthma [52]. For the role of Tr, they can also inhibit ILC2s in mouse asthma models via the production of IL-10 and TGF-β. The suppression of human ILC2s involves the same cytokines [53]. 

### 1.3. Cytokines at Different Stages Play Crucial Roles in the Pathogenesis of Asthma 

**Figure 2:**
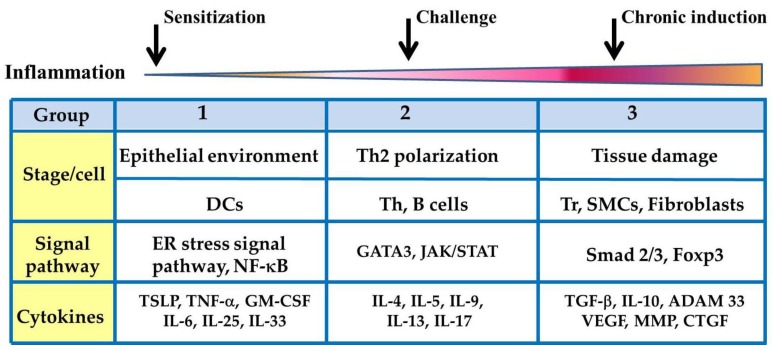
Three stages for cytokine therapeutic strategies of asthma.

According to the disease manifestation, three allergic development stages can be described: (1) the epithelial environment stage, (2) the Th2 polarization stage, and (3) the tissue damage stage. For the epithelial environment stage of asthma (allergic sensitization stage), air exposure to allergens induces the secretion of proinflammatory cytokines (Group 1) in the airway epithelium, such as TSLP, IL-6, IL-8, TNF-α, IL-25, IL-33, and GM-CSF. In this stage, therapeutic strategies focus on the suppression of inflammation. TSLP is a Th2-prone cytokine that induces a Th2 environment. It activates DCs, and the TSLP-activated DCs develop a Th2-prone microenvironment. Lung DCs are a heterogeneous cell population that contains conventional DCs (cDCs), plasmacytoid DCs (pDCs), and monocyte-derived DCs (moDCs) [43]. DCs are professional antigen-presenting cells that shape T helper cell polarization through different surface molecules and cytokines including IL-12, TGF-β, IL-6, IL-23, and IL-1β [54]. Stage 2 of asthma focuses on T cell priming and allergen restimulation. After the airway environment is polarized to the Th2-promoting condition, DCs may induce the differentiation of naive T cells into Th2 cells, which eventually leads to IgE production by B cells. After restimulation with a different antigen, Th0 cells may differentiate into Th2 cells, Th9 cells, or Th17 cells, and secrete different cytokines (Group 2), such as IL-4, IL-5, IL-9, IL-13, or IL-17, to activate eosinophils, basophil mast cells, or goblet cells. Eosinophil and mast cell degranulation at this stage plays a crucial role in airway hypersensitivity. In Stage 3 of asthma, local inflammation is induced by Group 3 cytokines such as TGF-βand IL-10 in the bronchus and lung, which leads to tissue repair initiation. TGF-β contributes to tissue repair and fibrosis. In addition, TGF-β and IL-10 contribute to regulatory cell development (Figure 2).

## 2. Therapeutic Drugs for Asthma

Clinically, the main treatments for asthma are reliever and controller medications [1]. The strategies for the prevention and alleviation of asthma are complicated. Relievers are divided into three categories as follows: (1) short-acting inhaled β2 agonist bronchodilators (e.g., salbutamol and terbutaline), (2) short-acting inhaled anticholinergics, and (3) low-dose inhaled corticosteroids (ICSs) plus formoterol. Controllers are divided into four categories as follows: (1) ICSs, (2) ICSs and long-acting β2 agonist bronchodilators, (3) chromones, and (4) leukotriene modifiers. Add-on controllers are categorized into four categories as follows: (1) systemic steroids, (2) long-acting inhaled anticholinergies, (3) anti-IgE, and (4) anti-IL-5 [1]. Corticosteroids are currently the most efficacious drugs used to control and treat asthma [55]. They can reduce the number of eosinophils, T lymphocytes, mast cells, and DCs during respiratory inflammation; inhibit proinflammatory cytokine production; and decrease the incidence of asthma and exercise-induced asthma [56,57,58]. However, the long-term overuse of systemic steroids may have many side effects, such as the inhibition of height growth and an increase in the risk of osteoporosis, adrenal insufficiency, and diabetes [59,60,61,62]. Moreover, there are different endotypes of asthma, and some of these can be treated well with steroids, but there are still many cases with treatment refractory asthma; therefore, novel therapies are needed. Salmeterol is a long-acting β2 agonist drug that reduces the severity of asthma in children by suppressing TSLP secretion in human bronchial ECs [63]. The anti-IgE antibody marks the beginning of a new era of monoclonal antibodies (MAbs) in the treatment of asthma. The humanized anti-IgE antibody omalizumab has been confirmed to improve the asthma symptom score, reduce the chances of acute asthma attacks, reduce the dosage of oral or inhaled glucocorticoid, and improve the quality of life of patients with asthma. Omalizumab also decreases airway wall thickening, decreases the percentage of sputum eosinophils, and increases forced expiratory volume in one second (FEV1) in asthma [64]. Omalizumab also reduces IgE-stimulated synthesis and the secretion of the proinflammatory cytokines IL-6, IL-8, TNF–α, and IL-4 by human airway smooth muscle cells (ASMCs) [65]. 

## 3. Clinical and Investigational Cytokine-Targeting Therapy for Asthma

Recently, cytokine-targeting biologics developed by clinicians have become potential therapy for asthma. In general, cytokine-targeting biologics for the treatment of asthma can be directly targeted through three mechanisms as follows: soluble receptors, anti-cytokine antibodies, and anti-cytokine receptor antibodies [66], and antisense approaches [67]. For three anti-asthmatic cytokine strategies, we summarized the different cytokines involved in each stage, and different stages target different cytokine candidates (Figure 2). 

The potential cytokine-targeting therapies for asthma are discussed as below (Table 1). Group 1 consists of an anti-TSLP antibody, anti-IL-33R antibody, anti-IL-33R antibody, anti-IL-25 antibody, and anti-IL-6 antibody, which are investigational drugs for asthma. TSLP assists natural helper cells in inducing corticosteroid resistance in patients with asthma [68]. The anti-TSLP antibody decreases sputum and blood eosinophils and reduces allergen-induced bronchoconstriction in patients with allergic asthma [69]. The anti-TSLP antibody also exerts preventive effects on airway structural changes for smooth muscle thickness in asthma [70]. The human anti-TSLP antibody tezepelumab has decreased the annualized rate of asthma attacks in patients with uncontrolled asthma who were already being treated with medium to high doses of inhaled glucocorticoids and long-acting β-agonists [70,71]. The IL-33 trap is a new antagonist of IL-33 that has been proven to inhibit allergic airway inflammation in an in vitro animal study [72]. AMG 282 and ANB020 has been developed in clinical trials on asthma, and it is a drug that targets soluble IL-33 [71]. The receptors for IL-33 are expressed on many cells involved in the allergic response, including TH2 cells, ILC2 cells, macrophages, hematopoietic stem cells, eosinophils, basophils, mast cells, and fibroblasts. The anti-IL-33R antibody and CNTO 7160 have been studied in asthma, but the final report has remained unpublished [71]. Additionally, the anti-IL-25 antibody has been studied. The anti-IL-25 antibody significantly reduced the levels of IgE, IL-5, and IL-13; goblet cell hyperplasia; and eosinophil infiltration, and prevented AHR in murine asthma models [73]. However, no human clinical study of the anti-IL-25 antibody has been performed. The anti-IL-6 antibody for granulocytic airway inflammation therapies in asthma has also been reported [74]. A large number of human clinical trials of anti-IL-6 antibody have been performed. Group 2 consists of the anti-IL-4Rα antibody, anti-IL-5 antibody, anti-IL-13 antibody, anti-IL-9 antibody, and anti-IL-17 antibody, which are the investigational drugs for asthma. The anti-IL-4Rα antibody is directed against IL-4Rα and blocks the IL-4 and IL-13 pathways; this asthma treatment is under development [75]. The human anti-IL-4Rα antibody dupilumab increases the forced exhalation volume in one second, and decreases severe exacerbations in patients with uncontrolled persistent asthma [75]. Patients who received dupilumab had better lung function, asthma control, and significantly lower rates of severe asthma exacerbation [76]. Dupilumab is still under investigation for use as an add-on controller in asthma treatment. The anti-IL-5 antibody now is an add-on controller for patients with severe asthma. The humanized anti-IL-5 antibody mepolizumab improves FEV1 and reduces the number of eosinophils in the sputum and blood in asthma [77]. The humanized anti-IL-5α antibody benralizumab showed significant decreases in oral glucocorticoid use and exacerbation rates compared with placebo [66]. The Food and Drug Administration of the United States approved mepolizumab and reslizumab as new anti-IL-5 therapies for the treatment of severe eosinophilic asthma [78]. There has been a report on anti-interleukin-5 receptor α monoclonal antibody as an add-on treatment for patients with severe, uncontrolled, eosinophilic asthma. Benralizumab significantly reduced annual exacerbation rates and was generally well tolerated for patients with severe, uncontrolled asthma with 300 cells per μL or greater of blood eosinophils [79]. Although the study treatments were not connected with any deaths, serious adverse events occurred in some patients (<1%). The Food and Drug Administration of the United States also approved benralizumab as a new therapy for severe asthma. The human anti-IL-13 antibody tralokinumab decreased the use of β-agonists and improved lung function, but no improvement in the Asthma Control Questionnaire 6 score was observed in moderate to severe asthma cases [80]. The humanized anti-IL-13 antibody lebrikizumab improved lung function and the rate of asthma exacerbations in patients with moderate to severe asthma [81]. The anti-IL-9 antibody inhibited the pulmonary infiltration of inflammatory cells and decreased the production of cytokines IL-5, IL-9, and IL-17 in murine asthma models [82]. However, the humanized anti-IL-9 antibody MEDI-528 did not decrease asthma exacerbation rates and did not improve Asthma Control Questionnaire 6 scores or FEV1 values [83]. The anti-IL-17 antibody decreased oxidative stress, pulmonary inflammation, and edema in animal models of asthma [84]. The human anti-IL-17 antibody brodalumab improved Asthma Control Questionnaire scores, with nominal significance noted only for the high-reversibility subgroup asthma [85]. However, a study of anti-IL17A (brodalumab) in adults with moderate-to-severe asthma showed no improvement in asthma control [85]. There are still no Group 3clinical drugs for human: anti-TGF-β cytokine is the one recent anti-cytokine antibody in this group. In an asthmatic animal model, anti-TGF-βAb treatment prevented the progression of airway remodeling following allergen challenge, even when was given in a therapeutic model [86]. However, a previous study reported that anti-TGF-β treatment had no effect on airway remodeling and exacerbated the eosinophilic infiltrate, which led to increased airway hyperreactivity to the house dust mite-induced allergic disease [65]. Further evaluation is warranted. For the output of selected clinical trials on antisense drugs related to inflammatory disorders, cytokine antisense approaches on asthma are focusing on GATA3 [87] and beta subunit (β(c)) of the IL-3, IL-5, and GM-CSF receptors and the chemokine receptor CCR3; [88] their outcomes are safe and can reduce in allergen-induced early-phase and late-phase asthmatic responses. 

## 4. Cytokine Immunomodulatory Effects of Natural Formula, Herbs, and Natural Compounds on Asthma

Many ancient countries have acquired knowledge regarding traditional herbal remedies. Natural formula, herbs, or compounds derived from plants have been found to alleviate asthma inflammation symptoms. Different groups of natural compounds according to their biosynthetic origin may be used as supplements for asthma prevention or therapy. According to the specific structures and bioeffects, anti-asthmatic bioactive compounds can be divided into five types: alkaloids, flavonoids, glycosides, polyphenols, and terpenoids [89]. Many studies have evaluated the immunomodulatory effect of these compounds by using the murine asthma model.

Herbal medicines and natural products are now used for integrative therapy and clinical drug development for asthma. In traditional Chinese medicine, treatment for asthma has been described earlier in Danxi’s Mastery of Medicine (1347 BC), but the formulas for asthma symptom relief, namely Xiao-Qing-Long-Tang, were described by Zhang Zhong-Jing in Shanghan Lun (219 BC). In this article, we summarize the regulation effects of herbal formulas on cytokines in the asthma model. Regarding formulas, Xiabai powder has been found to inhibit Group 1 cytokine (TNF-α, IL-1β, and IL-6) expression [90]. The antiasthma simplified herbal medicine intervention (ASHMI) alleviates asthma symptoms by modulating Group 1 cytokine (inhibition of TNF-α and IL-6) [91] and Group 2 cytokine (inhibition of IL-17, IL-13, IL-5, and IL-4, and enhancement of IFN-γ) expression [92,93]. The precursors of ASHMI, MSSM-002, inhibit Group 2 cytokines (the inhibition of IL-4, IL-5, IL-13, and GATA-2 and the enhancement of IFN-γ expression) to relieve asthma symptoms [93,94]. The modulators of Group 2 cytokine expression, such as the Sanao decoction, [95] Buzhong Yiqi decoction, [90] Shengfei Yuchuan decoction, [90] Wheeze-relief formula, [90] Wuwei Dilong decoction, [90] Bushen Yiqi decoction, [89] STA-1, [96] and modified Mai-Men-Dong-Tang [89] can be used in patients with asthma. Most formulas exert their beneficial effects by downregulating Group 2 cytokine expression, whereas only Xiabai powder was found to inhibit Group 1 cytokine expression [90]. Xiao-Qing-Long-Tang can regulate the expression of the cytokines in groups 1 to However, Xiao-Qing-Long-Tang exerts its effect only through the enhancement of CD4+ CD25 + Foxp3 + T cells and Foxp3; no data were found regarding its effect on Group 3 cytokine expression [97]. The cytokine inhibitory effects of herb extracts and major active compounds on asthma are presented in Table 2 [89,98,99,100,101,102,103,104,105,106,107,108,109,110,111,112,113,114,115,116,117,118,119,120,121,122,123,124,125,126,127,128,129,130,131,132,133,134,135,136]. For example, the herbs *Cordyceps sinensis*, *Thuja orientalis*, *Fritillaria thunbergii*, *Scutellaria baicalensis*, *Astragalus membranaceus*, *Curcuma longa*, and *Alstonia scholaris* show inhibitory effects on Group 1 cytokines, namely IL-1β, IL-6, and TNF-α. However, the herbs and their active components are more effective at inhibiting Group 2 cytokine expression than the other two cytokine groups. Group 3 cytokines, namely TGF-β1 and IL-10, can be inhibited by *Propolis*, *A. membranaceus* (Astragaloside IV), *Ligusticum wallichii*, and *Peucedanum praeruptorum* ((±)-praeruptorin A). *A. membranaceus* and Astragaloside IV inhibit the Group 1, 2, and 3 cytokines, which may mean that the herbs show multifunctional effects on the expression of proinflammatory cytokines. However, the herb extracts have complex mechanisms of action (MOA) compared with conventional drugs. The MOA of herbs may involve pro-inflammatory cytokine secretion through various molecular signaling pathways. Additionally, the quality and consistency of herbs may be difficult to control, which may limit the use of herbal extracts as integrative therapy for asthma.

Natural products can be divided into many types according to their molecular structures and different bioactivities. In this article, we discuss five major types: flavonoids, triterpenoids and glycosides (saponins), alkaloids, polyphenols, and other compounds, namely triptolide; they exhibit unique activity for pro-inflammatory cytokine expression in patients with asthma. The effect and mechanism of these compounds are provided in detail in Table 3 [100,106,107,116,126]. The target pathways of these compounds are still unclear. However, some immunomodulatory mechanisms have been clarified. Flavonoids are powerful antioxidants that inhibit chemical mediators initiating Th2-type cytokine synthesis, and they also inhibit other mechanisms that involve mast cells and basophils. Flavonoids block IL-4-induced signal transduction and influence the differentiation of T cells through the aryl hydrocarbon receptor [137]. The target signaling pathway affected by polyphenols is the NF-κB signaling pathway [138]. Polyphenols suppress T helper 2 activation and promote the development of regulatory T cells (Tr) [139]. Flavonoids can also modulate DC functions either by dampening MHC-II and the costimulatory molecule expression or by inhibiting cytokine production, thus hampering the antigen presentation process [140]. Triterpenoids and their glycosides. (saponin) also affect the NF-κB signaling pathway, and they function as anti-inflammatory agents [141]. Alkaloids affect STAT6 and the forkhead box P3 (Foxp3), NF-κB, and mitogen-activated protein kinase (MAPK) signaling pathways to modulate pro-inflammatory cytokine expression.

## 5. The Side Effect and Specific Outcomes in Asthma of Herbal Compounds

Most herbal formulas can alleviate AHR and late-phase inflammation for asthma. Especially, Sanao decoction and Xiao-Qing-Long-Tang can modulate the Gr 3 reaction. In the clinical trial, ASHMI, Ding Chuan Tang, STA-1, and Mai-Men-Dong-Tang have been evaluated; the outcomes are safe and improve lung function [186]. However, *Ephedra sinica* derivated from Shengfei Yuchuan decoction, Wuwei Dilong Decoction, and Xiao-Qing-Long-Tang have side effects on the cardiovascular system [90]. The bioeffect of a single herb for asthma treatment has been evaluated by the OVA animal model; otherwise, some study used an allergen-animal model to evaluate its bioeffect. Among them, *Tripterygium polyglycosid* and a major active component, triptolide, have side effects included hematologic abhormalities, gastriointestinal intolerance, infection, and infertility [84]. In the markets, natural products claimed multiple contents for multiple bioeffects, and the knowledge from biocompounds will apply to the new drug development field. We conclude that the natural component divided in Group 1 can provide preventive effects at the early stage of asthma development; the natural components divided into groups 2 and 3 have therapeutic potency. However, balance is key: too much of any of them has no benefit for prevention or treatment at all.

## 6. Conclusions

The pathophysiology of asthma disorders is complex. Cytokines play a crucial role in the immune system and inflammatory responses in asthma. Many inflammatory cytokines are involved in innate and adaptive immunity in asthma. Therefore, anti-cytokine antibodies/anti-cytokine receptor antibodies are potential therapy for patients who do not respond to standard treatments. Except for asthma, the efficacy and safety have been proven for cardiovascular, cancerous, respiratory, hematology, autoimmune and infectious diseases. As of March 2017, the Food and Drug Administration (FDA) has approved approximately 60 therapeutic MAbs until March 2017 that are currently under evaluation in various phases of clinical trials. Adverse reactions have been reported, including immune regulation disorder, other immune-related adverse reactions such as dermatologic, gastrointestinal, and endocrine, and reactions related to alterations in the immune balance, including undesired effects related to the target antigens and cytokine release syndrome [187]. No single cytokine is responsible for the entire pathogenesis of asthma. This is the challenging aspect of MAbs for asthma therapy. Therefore, the evaluation of anti-cytokine antibodies/anti-cytokine receptor antibodies for different phenotypes of asthma is essential.

In accordance with ancient pharmacopoeias, many medicinal plants show immunomodulatory potential and anti-asthmatic effects from the beginning of allergen sensitization to Th2 polarization, pulmonary inflammation, and fibrosis. An evidence-based study of natural medicinal herbs in treating asthma suggested that in addition to alleviating airway syndromes, many natural products have immunomodulatory effects, including modulating inflammatory cytokine expression and regulating the activity of inflammatory cells. Further research is warranted to explore the detailed immunomodulatory molecular mechanisms of these natural compounds to elucidate the in vitro and in vivo mechanisms of these active ingredients and ascertain their therapeutic management in asthma. Although these natural compounds, which have existed for a long period of time, can be used for preventive or therapeutic purposes, the precise dosage of natural medicinal compounds for patients with asthma still needs further evaluation. Traditional herbal products, especially formulas and single herbs, are widely used in asia. Although many studies have proved their biofunction, the standardized preparation, dosage use, and drugs interaction, and other side effects all need more effort to achieve safe delivery and efficacy [188]. 

## Figures and Tables

**Table 1:** Recent clinical and investigational anti-cytokine Ab for asthma therapy.

Group	Anti-Cytokine Ab	Drug
Group 1	Anti-TSLP Ab	Tezepelumab (Phase 3 clinical trial)
	Anti-IL-6 Ab	N/A
	Anti-IL-25 Ab	N/A
	Anti-IL-33 Ab	AMG 282 (Phase 1 clinical trial) ANB020 (Phase 2 clinical trial)
	Anti-IL-33R Ab	CNTO 7160 (Phase 1 clinical trial)
Group 2	Anti-IL-4Rα Ab	Dupilumab (Phase 3 clinical trial)
	Anti-IL-5 Ab	Mepolizumab (US FDA approved) Reslizumab (US FDA approved)
	Anti-IL-5Rα Ab	Benralizumab (US FDA approved)
	Anti-IL-9 Ab	MEDI-528 (Phase 2 clinical trial)
	Anti-IL-13Ab	Tralokinumab (Phase 3 clinical trial) Lebrikizumab (Phase 3 clinical trial)
	Anti-IL-17 Ab	Brodalumab (Phase 2 clinical trial)
Group 3	Anti-TGF-β Ab	N/A

FDA: Food and Drug Administration, IL: interleukin, N/A: not available, TSLP: thymic stromal lymphopoietin, US: United States. Transforming Growth Factor Beta: TGF-β.

**Table 2:** Relationship of cytokines with single herb and components. GM-CSF: granulocyte-macrophage colony-stimulating factor, IgE: immunoglobulin E, TNF-α: tumor necrosis factor-α.

Group	Components	Cytokines *	Ref.
**Group 1**			
*Cordyceps sinensis*	CS-19-22 fraction	IL-1β, TNF- α, IL-6, IL-10 (–) (LPS-activated BALF cells)	[98]
*Astragalus membranaceus*	Astragaloside IV	IL-1β, TNF- α, GM-CSF (–) (Der p 1 activated human blood eosinophils)	[99]
*Curcuma longa*	Curcumin	IL-1β, TNF- α, IL-6, IL-2 (–) (DRA-challenged mice/ LPS-stimulated macrophages)	[100,101]
*Thuja occidentalis*	Extract	IL-6, TNF-α (–) (LPS-stimulated macrophages)	[102]
*Fritillaria thunbergii*	Extract	IL-6, TNF-α (–) (Human mast cell line-1 for childhood asthma)	[89]
*Scutellaria baicalensis*	Extract	TNF-α (–) (compound 48/80-induced HMC-1 cells)	[103]
*Alstonia scholaris*	Total alkaloid	TNF-α, (–) (LPS-induced airway inflammation in rats)	[104]
**Group 2**			
*Astragalus membranaceus*	Extract	IL-4, IL-5, IL-13 (–), IFN-γ (+)	[105,106]
	Astragaloside IV	IFN-γ (+), IL-4, IL-5, IL-13 (–)	[107,108]
*Asparagus cochinchinensis*	Saponin-enriched extract	IL-4, IL-13 (–)	[109]
*Peucedanum praeruptorum*	Coumarins	IL-4, IL-5, IL-13 (–), IL-10, IFN-γ (+)	[110]
	(±)-praeruptorin A	IL-4, IL-5, IL-12, IL-13 (–)	[111,112]
*Victis fructus*	Pyranopyran-1, 8-dione	IL-4, IL-5, IL-13 (–) (Cockroach allergen-induced mice)	[113]
*Glycyrrhiza uralensis*	Isoliquiritigenin 7, 4’-DHF, liquiritigenin	IL-4, IL-5, IL-13, GATA-3 (–), IFN-γ (+) (effector memory Th2 cells D10 and OVAsensitized/challenged mice)	[114]
*Ilex chineses*	Protocatechuic acid	IL-4, IL-5, IL-13 (–)	[115]
*Rheum officinale*	Emodin	IL-4, IL-5, IL-13 (–)	[116]
*Lithonspermum erythrorthizon*	Shikonin	IL-4, IL-5, IL-13, TNF-α (–) (OVA/TSLP-induced BM-DCs maturation)	[117]
*Ganoderma tsugae*	Triterpenoid-rich extracts	IL-4, IL-5 (–)	[118]
*Thuja orientalis*	Extract	IL-4, IL-5, IL-13 (–) (LPS-stimulated macrophages)	[102]
*Ligusticum wallichi*	Ligustrazine	IL-4, IL-5, IL-13, IL-17, TNF-α (–)	[119]
*Armeniacae amarum*	Water extract	IL-4 (–)	[120]
*Morus alba*	Kuwanon G	IL-4, IL-5, IL-13 (–)	[121]
*Pinellia ternate*	Water extract	IL-4, IL-5, IL-13, TNF-α (–)	[122,123]
*Mentha haplocalyx*	Ethanol extract	IL-5 (–)	[124]
*Platycodon grandiflorum*	Water extract	IL-4, IFN-γ, IL-5, IL-13, TNF-α (–)	[125]
	Saponins	IL-4, TNF-α (–) (IgE antibody-induced RBL-2H3 cell)	[126]
*Scutellaria baicalensis*	Skullcapflavone II	IL-4, TNF-β1 (–)	[127]
*Echinodarus scaber*	Hydroethanolic extract	IL-4, IL-5, IL-13 (–)	[128]
*Propolis*	Caffeic acid phen-ethyl ester	IL-4, IL-5, TNF-α (–)	[129]
*Tripterygium polyglycosid*	Extract	IL-5 (–)	[130]
	Triptolide	IL-5, IL-12, TGF-β1 (–) (LPS-stimulated MPM and human MDC)	[131,132]
*Propolis*		IL-10, IFN-γ, IL-5, IL-6 (–),	[133]
*Cordyceps sinensis*	CS-19-22 fraction	IFN-γ, IL-12 (+) (LPS-activated BALF cells)	[98]
*Curcuma longa*	FLLL31 (derivative of curcumin)	IL-17 (–) (DRA-challenged mice and LPS-stimulated macrophages	[101]
*Anoectochilus formosanus*	Extract	IL-4, TNF-α (–), IFN-γ, IL-12 (+)	[134]
*Gynostemma pentaphyllum*	Extract	IFN-γ (+)	[135]
**Group 3**			
*Astragalus membranaceus*	Astragaloside IV	TGF-β1 (–), IL-10 (+)	[136]
*Ligusticum wallichii*	Ligustrazine	IL-10 (+)	[119]
*Peucedanum praeruptorum*	(±)-Praeruptorin A	TGF-β1 (–)	[111]
*Tripterygium polyglycosid*	Triptolide	TGF-β1 (–)	[132]

* The cytokine regulation in Table 2 are measured by OVA animal model, the special results measured by other cell or animal model were noted in the table. LPS: lipopolysachride; BALF: Bronchoalveolar lavage fluid; DRA: triple allergens (dust-mite, ragweed, and Aspergillus); OVA: Ovalbumin; MPM: malignant pleural mesothelioma and MDC: myeloid dendritic cells (mDC)).

**Table 3:** Relationship of cytokines with the compounds. NF-κB: nuclear factor-κB.

Type	Compound	Cytokine *	Mechanisms	Ref.
Flavonoids	Chrysin	Gr1: IL-1β, IL-6 (–) Gr2: IL-4, TNF-α (–)	Inhibition of the NF-κB signaling pathway and caspase-1	[142]
	Kaempferol	Gr2: IL-4, IL-5, IL-13 (–) (A23187-stimulated KU812 cells)	Inhibition of the NF-κB signaling pathway	[143,144]
	Fisetin	Gr2: IL-4, IL-5, IL-13, TNF-α (–) (A23187-stimulated KU812 cells) Gr 1: IFN-γ, IL-8, IL-1β (–)	Inhibition of the MyD88 and NF-κB signaling pathways	[144,145,146]
	Quercetin	Gr1: IL-1β, IL-6 (–) (A23187-stimulated KU812 cells) Gr2: IL-4, IL-5, TNF-α, IFN-γ (–) Gr3: IL10 (+) (BV-2 LPS-stimulated microglia cells)	Inhibition of protein kinase C θ phosphorylation inhibition of the NF-κB signaling pathway	[144,146,147,148]
	Skullcap-flavone II	Gr2: IL-4, IL-5, IL-13 (–) Gr3: TGF-β1 (–)	Acting on TGF-β1/Smad signaling pathways	[127]
	Morin	Gr1: IL-1β, IL-6 (–) Gr2: TNF-α, IL-4, IL-13 (–)	up-regulated SUMF2 mRNA expression and down-regulated Leukotriene B4 receptor 2 (BLT2)/NF-kB mRNA expression	[149]
	Myricetin	Gr1: IL-6, IL-8, TNF-α (–) (Human umbilical cord blood-derived cultured mast cells)	Inhibition of protein kinase C θ phosphorylation	[147]
	Cyanidin	Gr1: IL-17A (–)	Inhibition of the IL-17A/IL-17RA interaction	[150]
	Tangeretin	Gr1: IL-6 (–) Gr2: IFN-γ (+), IL-4, IL-5, IL-13, IL-17A (–)	Modulate PI3K/Akt and Notch signaling and Th2/Th1 and Th17 cytokine levels	[151]
	Rutin	Gr2: IL-4, IL-5, IL-13, IL-17A (–), IFN-γ (+) Gr3:IL-10 (+)	Inhibition of the NF-κB signaling pathway	[152]
	Kaempferol glycosides	Gr2: IL-5, IL-13 (–)	Inhibition of IL-4-induced transcription factor STAT6 activation	[143]
	Baicalin	Gr1: IL-6 (–) Gr2: IL-17A (–) Gr3: IL-10 (+)	Suppression of STAT3 expression and promoted FOXP3 expression	[153]
	Naringenin	Gr2: IL-4, IL-13 (–)	Inhibition of the NF-κB signaling pathway	[146,154]
	Esculento-side A	Gr2: IL-4, IL-5, IL-13 (–)	Nrf-2 activation	[155]
	Genistein and Daidzein	Gr1: IL-1β, TNF-α (–)	Inhibition of STAT-1 and NF-κB pathways	[156]
	Pinitol	Gr2: IFN-γ (+), IL-4, IL-5 (–)	Blocking the transcription factor GATA binding protein 3 (GATA 3)	[157]
	Flavocoxid	Gr2: IL-13 (–)	-	[158]
	Apigenin	Gr1: IL-6, TNF-α (–) Gr2: IL-17A (–), IL-4 (–)	Blocking the transcription factor GATA 3	[146,159]
	Luteolin-7-*O*-glucoside	Gr2: IL-4, IL-5, IL-13 (–)	Downregulation of T helper 2 cytokine transcript	[146,160]
Triterpenoid and glycosides	Astragaloside IV	Gr2: IL-4 (–), IFN-γ (+) Gr3: IL-10 (+)	Inhibition of the synthesis of GATA-3-encoding mRNA and protein in addition to increasing the synthesis of T-bet-encoding mRNA and protein in both lung tissues and CD4+ T cells	[107,108]
	α-Hederin	Gr2:IL-13, IL-17A (–), IL-2 (+)	Th1 cells (increases the Th1/Th2 ratio)	[161,162]
	Diosgenin	Gr1: TNF-α, IL1-β, IL-6 (–)	Enhancing the expression of glucocorticosteroid receptors, SLPI, GILZ, and MKP-1, and inhibiting the expression of HSP70	[163]
	Jujuboside B	Gr2: IL-4, IL-5 (–)	-	[164]
	Ganoderic acid C1	Gr1: TNF-α (–) (RAW264.7 cells and peripheral bloodmononuclear cells (peripheral blood mononuclear cells; PBMCs) from asthma patients)	Downregulation of NF-κB expression, and partial suppression of MAPK and AP-1 signaling pathways	[165]
	Lupeol	Gr1: TNF-α, IL-1β (–) Gr2: IL-4, IL-5, IL-13 (–)	A mechanism distinct of glucocorticoids,	[166,167]
	Boswellic acid	Gr2: IL-4, IL-5, IL-13 (–)	Decreasing the expression of pSTAT6 and GATA-3	[168]
	Celastrol	Gr1: TNF-α, IL-1β (–) (LPS-stimulated BV-2 cells)	Inhibition of extracellular signal-regulated kinase 1 and 2 (ERK1/2) phosphorylation and NF-κB activation	[169]
	B-Escin	Gr2: IL-5, IL-13 (–)	-	[170]
	Lupeol	Gr2: IL-4, IL-5, IL-13 (–) (LPS-treated marcophages)	-	[167]
			
Alkaloids	Sinomenine	Gr2: IL-4, IL-5, IL-13 (–) Gr3: TGF-β (–)	Inhibition of TH2 immune response, apoptosis of airway ECs and airway remodeling	[171]
	Chelidonine	Gr2:, IL-4, IL-13 (–)	STAT6 and Foxp3 pathways	[172]
	Protostemonine	Gr2: IL-4, IL-5, IL-13, IL-33 (–) (dust mites, ragweed and aspergillus-induced asthma)	Inhibition of STAT6, KLF4, and IRF4	[173]
	Ligustrazine	Gr2: IL-4 (–), IFN-γ (+)	Modulating key master switches GATA-3 and T-bet	[174]
	Ambroxol	Gr2: IL-4, L-13 (–)	Inhibiting IgE-dependent basophil mediator release and p38 MAPK activity	[175]
	Berberine	Gr1: IL-1β, IL-6 (–) Gr2: IL-4, IL-5, IL-13, IL-17 (–)	Inhibition of the NF-κB signaling pathway	[176]
Polyphenols	Epigallocatechin-3-gallate	Gr1: TNF-α (–) Gr2: IL-5 (–) (Toluene diisocyanate-induced asthma model)	Activation of the 5’ AMP-activated protein kinase (AMPK) signaling pathway	[177]
	Curcumin	Gr1: TNF-α, IL-1, IL-6 (–) Gr2: IL-2, IL-12 (–)	Inhibition of the NF-κB signaling pathway	[101]
	Ellagic acid	Gr2: IL-4, IL-5, IL-13 (–)	Inhibition of the NF-κB signaling pathway	[178,179]
	Resveratrol	Gr2: IL-4, IL-5 (–)	Inhibition of the NF-κB signaling pathway	[180]
	Apocynin	Gr1: TNF-α (–) Gr2: IL-4, IL-5, IL-12, IL-13 (–)	Inhibition of the NF-κB signaling pathway	[181]
Others	Triptolide	Gr2: IL-2 (+) Gr3: TGF-β1 (–)	TGF-β1/Smad pathway	[182]
	Andrographolide	Gr2: IL-4, IL-5, IL-13 (–)	Inhibition of the NF-κB signaling pathway	[183]
	Honokiol	Gr1: TNF-α, IL-6 (–) Gr2: IL-12, IFN-γ (+), IL-13, IL-17(–) Gr3: IL-10, TGF-β (+)	γ-Aminobutyric acid type A-dependent manner	[184]
	Thymoquin-one	Gr2: IL-4 (–), IFN-γ (+)	-	[185]
	Shikonin	Gr1: TNF-α (–) Gr2: IL-4, IL-5, IL-13 (–) (OVA + TSLP-induced BM-DC maturation, OVA-sensitized/challenged mice)	-	[117]

Gr1: Group 1; Gr2: Group 2; Gr3: Group 3; (+): upregulation; (−): suppression; −: no mentioned the detailed signal pathway. * The cytokine regulation in Table 3 are measured by OVA animal model, the special results measured by other cell or animal model were noted in the table.

## Abbreviations

dendritic cells (DCs); epithelial cells (ECs); interleukin (IL); granulocyte-macrophage colony-stimulating factor (GM-CSF); tumor necrosis factor-α (TNF-α); airway hyperresponsiveness (AHR); thymic stromal lymphopoietin (TSLP); innate lymphoid cells (ILC2); immunoglobulin E (IgE); nuclear factor-κB (NF-κB); antigen presenting cells (APCs); histocompatibility complex (MHC); T helper type 2 (Th2); vascular cell adhesion protein 1 (VCAM-1); regulatory T cells (Tr) ; interferon–γ (IFN-γ); transforming growth factor-beta (TGF-β); a disintegrin and metalloproteinase-33 (ADAM-33); connective tissue growth factor (CTGF); matrix metalloproteinase-9 (MMP-9); major histocompatibility complex (MHC); macophage inflammatory protein 1 alpha (MIP-1α); monocyte chemoattractant protein 1 (MCP1); regulated on activation, normal T cell expressed and secreted (RANTES); intercellular adhesion molecule (ICAM); janus kinase/signal transducers and activators of transcription (JAK–STAT); RAR-related orphan receptor gamma (RORγt); RAR-related orphan receptor alpha (RORα); interferon regulatory factor 4 (IRF-4); forced expiratory volume in one second (FEV1); airway smooth muscle cells (ASMCs); forkhead box P3 (Foxp3); mitogen-activated protein kinase (MAPK); Food and Drug Administration (FDA) monoclonal antibodies (MAbs).

## References

[1] Kim D.Y., Park J.W., Jeoung D., Ro J.Y. (2009). Celastrol suppresses allergen-induced airway inflammation in a mouse allergic asthma model. Eur. J. Pharmacol..

[2] Masoli M., Fabian D., Holt S., Beasley R. (2004). The global burden of asthma: executive summary of the GINA Dissemination Committee Report. Allergy.

[3] Soriano J.B., Abajobir A.A., Abate K.H., Abera S.F., Agrawal A., Ahmed M.B., Aichour A.N., Aichour I., Aichour M.T.E., Alam K. (2017). A Global, regional, and national deaths, prevalence, disability-adjusted life years, and years lived with disability for chronic obstructive pulmonary disease and asthma, 1990–2015: A systematic analysis for the Global Burden of Disease Study. Lancet Respirat. Med..

[4] Loftus P.A., Wise S.K. (2016). Epidemiology of asthma. Curr. Opin. Otolaryngol. Head Neck Surg..

[5] Serebrisky D., Wiznia A. (2019). Pediatric Asthma: A Global Epidemic. Ann. Glob. Health.

[6] Akinbami L.J., Moorman J.E., Liu X. (2011). Asthma Prevalence, Health Care Use, and Mortality.

[7] Centers for Disease Control and Prevention (CDC) (2011). Prevention, Vital signs: Asthma prevalence, disease characteristics, and self-management education: United States, 2001–2009. MMWR. Morb. Mortal. Wkly. Rep..

[8] Lin S.C., Lin H.W., Chiang B.L. (2017). Association of croup with asthma in children: A cohort study. Medicine.

[9] Cohn L., Elias J.A., Chupp G.L. (2004). Asthma: Mechanisms of disease persistence and progression. Annu. Rev. Immunol..

[10] Peebles R.S., Aronica M.A. (2019). Proinflammatory Pathways in the Pathogenesis of Asthma. Clin. Chest Med..

[11] Boulet L.P., Sterk P.J. (2007). Airway remodelling: The future. Eur. Respir. J..

[12] Mauad T., Bel E.H., Sterk P.J. (2007). Asthma therapy and airway remodeling. J. Allergy Clin. Immunol..

[13] Jeffery P.K. (2001). Remodeling in asthma and chronic obstructive lung disease. Am. J. Respir. Crit. Care Med..

[14] Bai T.R., Cooper J., Koelmeyer T., Pare P.D., Weir T.D. (2000). The effect of age and duration of disease on airway structure in fatal asthma. Am. J. Respir. Crit. Care Med..

[15] Royce S.G., Lim C.X.F., Patel K.P., Wang B., Samuel C.S., Tang M.L.K. (2014). Intranasally administered serelaxin abrogates airway remodelling and attenuates airway hyperresponsiveness in allergic airways disease. Clin. Exp. Allergy.

[16] An S.S., Bai T.R., Bates J.H., Black J.L., Brown R.H., Brusasco V., Chitano P., Deng L., Dowell M., Eidelman D.H. (2007). Airway smooth muscle dynamics: A common pathway of airway obstruction in asthma. Eur. Respir. J..

[17] Holgate S.T. (2012). Innate and adaptive immune responses in asthma. Nat. Med..

[18] McWilliam A.S. (1994). Rapid dendritic cell recruitment is a hallmark of the acute inflammatory response at mucosal surfaces. J. Exp. Med..

[19] Lambrecht B.N., Hammad H. (2012). The airway epithelium in asthma. Nat. Med..

[20] Evans S.E., Xu Y., Tuvim M.J., Dickey B.F. (2010). Inducible Innate Resistance of Lung Epithelium to Infection. Annu. Rev. Physiol..

[21] Lin Y.L., Chen S.H., Wang J.Y. (2016). Critical role of IL-6 in dendritic cell-induced allergic inflammation of asthma. J. Mol. Med..

[22] Liew F.Y., Girard J.-P., Turnquist H.R. (2016). Interleukin-33 in health and disease. Nat. Rev. Immunol..

[23] Kouzaki H., Tojima I., Kita H., Shimizu T. (2013). Transcription of Interleukin-25 and Extracellular Release of the Protein Is Regulated by Allergen Proteases in Airway Epithelial Cells. Am. J. Respir. Cell Mol. Boil..

[24] Andreakos E., Papadopoulos N.G. (2014). IL-25: The Missing Link Between Allergy, Viral Infection, and Asthma?. Sci. Transl. Med..

[25] Lin S.-C., Cheng F.-Y., Liu J.-J., Ye Y.-L. (2018). Expression and Regulation of Thymic Stromal Lymphopoietin and Thymic Stromal Lymphopoietin Receptor Heterocomplex in the Innate–Adaptive Immunity of Pediatric Asthma. Int. J. Mol. Sci..

[26] Wong C.K., Hu S., Cheung P.F., Lam C.W. (2010). Thymic stromal lymphopoietin induces chemotactic and prosurvival effects in eosinophils: Implications in allergic inflammation. Am. J. Respir. Cell Mol. Biol..

[27] Comeau M.R., Ziegler S.F. (2010). The influence of TSLP on the allergic response. Mucosal. Immunol..

[28] Wang Y.-H., Liu Y.-J., Wang Y.-H., Liu Y.-J., Wang Y., Liu Y. (2009). Thymic stromal lymphopoietin, OX40-ligand, and interleukin-25 in allergic responses. Clin. Exp. Allergy.

[29] Nakajima H., Iwamoto I., Tomoe S., Matsumura R., Tomioka H., Takatsu K., Yoshida S. (1992). CD4+ T-lymphocytes and interleukin-5 mediate antigen-induced eosinophil infiltration into the mouse trachea. Am. Rev. Respir. Dis..

[30] Chung K.F., Barnes P.J. (1999). Cytokines in asthma. Thorax.

[31] Masinovsky B., Urdal D., Gallatin W.M. (1990). IL-4 acts synergistically with IL-1 beta to promote lymphocyte adhesion to microvascular endothelium by induction of vascular cell adhesion molecule-1. J. Immunol..

[32] Bossios A., Sjostrand M., Dahlborn A.K., Samitas K., Malmhall C., Gaga M., Lotvall J. (2010). IL-5 expression and release from human CD34 cells in vitro; ex vivo evidence from cases of asthma and Churg-Strauss syndrome. Allergy.

[33] Townsend M.J., Fallon P.G., Matthews D.J., Smith P., E Jolin H., McKenzie A.N. (2000). IL-9-Deficient Mice Establish Fundamental Roles for IL-9 in Pulmonary Mastocytosis and Goblet Cell Hyperplasia but Not T Cell Development. Immun..

[34] Fajt M.L., Wenzel S.E. (2017). Development of New Therapies for Severe Asthma. Allergy, Asthma Immunol. Res..

[35] Al-Alawi M., Hassan T., Chotirmall S.H. (2014). Transforming growth factor beta and severe asthma: A perfect storm. Respir. Med..

[36] Lin S.C., Chou H.C., Chiang B.L., Chen C.M. (2017). CTGF upregulation correlates with MMP-9 level in airway remodeling in a murine model of asthma. Arch. Med. Sci..

[37] Neill D.R., Wong S.H., Bellosi A., Flynn R.J., Daly M., Langford T.K.A., Bucks C., Kane C.M., Fallon P.G., Pannell R. (2010). Nuocytes represent a new innate effector leukocyte that mediates type-2 immunity. Nature.

[38] Price A.E., Liang H.-E., Sullivan B.M., Reinhardt R.L., Eisley C.J., Erle D.J., Locksley R.M. (2010). Systemically dispersed innate IL-13–expressing cells in type 2 immunity. Proc. Natl. Acad. Sci..

[39] Ito T., Liu Y.J., Arima K. (2012). Cellular and molecular mechanisms of TSLP function in human allergic disorders—TSLP programs the” Th2 code” in dendritic cells. Allergol. Int..

[40] Kurowska-Stolarska M., Kewin P., Murphy G., Russo R.C., Stolarski B., Garcia C.C., Komai-Koma M., Pitman N., Li Y., Niedbala W. (2008). IL-33 induces antigen-specific IL-5+ T cells and promotes allergic-induced airway inflammation independent of IL-4. J. Immunol..

[41] Elder M.J., Webster S.J., Williams D.L., Gaston J.S., Goodall J.C. (2016). TSLP production by dendritic cells is modulated by IL-1beta and components of the endoplasmic reticulum stress response. Eur. J. Immunol..

[42] Schuliga M. (2015). NF-kappaB Signaling in Chronic Inflammatory Airway Disease. Biomol..

[43] Kool M., Van Loo G., Waelput W., De Prijck S., Muskens F., Sze M., Van Praet J., Branco-Madeira F., Janssens S., Reizis B. (2011). The Ubiquitin-Editing Protein A20 Prevents Dendritic Cell Activation, Recognition of Apoptotic Cells, and Systemic Autoimmunity. Immun..

[44] Liu T., Zhang L., Joo D., Sun S.C. (2017). NF-kappaB signaling in inflammation. Signal Transduct. Target. Ther..

[45] Paul A.G.A., Muehling L.M., Eccles J.D., Woodfolk J.A. (2019). T cells in severe childhood asthma. Clin. Exp. Allergy.

[46] Wan Y.Y. (2014). GATA3: A master of many trades in immune regulation. Trends Immunol..

[47] Shrine N., Portelli M.A., John C., Soler Artigas M., Bennett N., Hall R., Lewis J., Henry A.P., Billington C.K., Ahmad A. (2019). Moderate-to-severe asthma in individuals of European ancestry: A genome-wide association study. Lancet Respir. Med..

[48] Vale K. (2016). Targeting the JAK-STAT pathway in the treatment of ‘Th2-high’ severe asthma. Futur. Med. Chem..

[49] Pernis A.B., Rothman P.B. (2002). JAK-STAT signaling in asthma. J. Clin. Investig..

[50] Wisniewski J.A., Muehling L.M., Eccles J.D., Capaldo B.J., Agrawal R., Shirley D.A., Patrie J.T., Workman L.J., Schuyler A.J., Lawrence M.G. (2018). TH1 signatures are present in the lower airways of children with severe asthma, regardless of allergic status. J. Allergy Clin. Immunol..

[51] Redington A.E., Madden J., Frew A.J., Djukanović R., Roche W.R., Holgate S.T., Howarth P.H. (1997). Transforming Growth Factor- β 1 in Asthma. Am. J. Respir. Crit. Care Med..

[52] Moffatt M.F., Gut I.G., Demenais F., Strachan D.P., Bouzigon E., Heath S., Von Mutius E., Farrall M., Lathrop M., Cookson W.O. (2010). A Large-Scale, Consortium-Based Genomewide Association Study of Asthma. New Engl. J. Med..

[53] Rigas D., Lewis G., Aron J.L., Wang B., Banie H., Sankaranarayanan I., Galle-Treger L., Maazi H., Lo R., Freeman G.J. (2017). Type 2 innate lymphoid cell suppression by regulatory T cells attenuates airway hyperreactivity and requires inducible T-cell costimulator-inducible T-cell costimulator ligand interaction. J. Allergy Clin. Immunol..

[54] A Patente T., Pelgrom L.R., Everts B. (2019). Dendritic cells are what they eat: how their metabolism shapes T helper cell polarization. Curr. Opin. Immunol..

[55] Hoch H.E., Szefler S.J. (2016). Intermittent steroid inhalation for the treatment of childhood asthma. Expert Rev. Clin. Immunol..

[56] Horvath G., Wanner A. (2006). Inhaled corticosteroids: effects on the airway vasculature in bronchial asthma. Eur. Respir. J..

[57] Barnes P.J. (2010). Inhaled Corticosteroids. Pharmaceuticals.

[58] Gotshall R.W. (2002). Exercise-Induced Bronchoconstriction. Drugs.

[59] Raissy H.H., Blake K. (2013). Does Use of Inhaled Corticosteroid for Management of Asthma in Children Make Them Shorter Adults?. Pediatr. Allergy, Immunol. Pulmonol..

[60] Chee C., Sellahewa L., Pappachan J.M. (2014). Inhaled Corticosteroids and Bone Health. Open Respir. Med. J..

[61] Sannarangappa V., Jalleh R. (2014). Inhaled Corticosteroids and Secondary Adrenal Insufficiency. Open Respir. Med. J..

[62] Egbuonu F., A Antonio F., Edavalath M. (2014). Effect of Inhaled Corticosteroids on Glycemic Status. Open Respir. Med. J..

[63] Harada M., Hirota T., Jodo A.I., Hitomi Y., Sakashita M., Tsunoda T., Miyagawa T., Doi S., Kameda M., Fujita K. (2011). Thymic Stromal Lymphopoietin Gene Promoter Polymorphisms Are Associated with Susceptibility to Bronchial Asthma. Am. J. Respir. Cell Mol. Boil..

[64] Hoshino M., Ohtawa J. (2012). Effects of Adding Omalizumab, an Anti-Immunoglobulin E Antibody, on Airway Wall Thickening in Asthma. Respir..

[65] Roth M., Tamm M. (2010). The effects of omalizumab on IgE-induced cytokine synthesis by asthmatic airway smooth muscle cells. Ann. Allergy, Asthma Immunol..

[66] Nair P., Wenzel S., Rabe K.F., Bourdin A., Lugogo N.L., Kuna P., Barker P., Sproule S., Ponnarambil S., Goldman M. (2017). Oral Glucocorticoid-Sparing Effect of Benralizumab in Severe Asthma. New Engl. J. Med..

[67] Potaczek D.P., Garn H., Unger S.D., Renz H. (2016). Antisense molecules: A new class of drugs. J. Allergy Clin. Immunol..

[68] Kabata H., Moro K., Fukunaga K., Suzuki Y., Miyata J., Masaki K., Betsuyaku T., Koyasu S., Asano K. (2013). Thymic stromal lymphopoietin induces corticosteroid resistance in natural helper cells during airway inflammation. Nat. Commun..

[69] Gauvreau G.M., O’Byrne P.M., Boulet L.P., Wang Y., Cockcroft D., Bigler J., FitzGerald J.M., Boedigheimer M., Davis B.E., Dias C. (2014). Effects of an anti-TSLP antibody on allergen-induced asthmatic responses. N. Engl. J. Med..

[70] Lin S.-C., Chou H.-C., Chen C.-M., Chiang B.-L. (2018). Anti-thymic stromal lymphopoietin antibody suppresses airway remodeling in asthma through reduction of MMP and CTGF. Pediatr. Res..

[71] Lawrence M.G., Steinke J.W., Borish L. (2018). Cytokine-targeting biologics for allergic diseases. Ann. Allergy, Asthma Immunol..

[72] Holgado A., Braun H., Van Nuffel E., Detry S., Schuijs M.J., Deswarte K., Vergote K., Haegman M., Baudelet G., Haustraete J. (2019). IL-33trap is a novel IL-33-neutralizing biologic that inhibits allergic airway inflammation. J. Allergy Clin. Immunol..

[73] Ballantyne S.J., Barlow J.L., Jolin H.E., Nath P., Williams A.S., Chung K.F., Sturton G., Wong S.H., McKenzie A.N. (2007). Blocking IL-25 prevents airway hyperresponsiveness in allergic asthma. J. Allergy Clin. Immunol..

[74] Chu D.K., Al-Garawi A., Llop-Guevara A., A Pillai R., Radford K., Shen P., Walker T.D., Goncharova S., Calhoun W.J., Nair P. (2015). Therapeutic potential of anti-IL-6 therapies for granulocytic airway inflammation in asthma. Allergy, Asthma Clin. Immunol..

[75] Wenzel S., Castro M., Corren J., Maspero J., Wang L., Zhang B., Pirozzi G., Sutherland E.R., Evans R.R., Joish V.N. (2016). Dupilumab efficacy and safety in adults with uncontrolled persistent asthma despite use of medium-to-high-dose inhaled corticosteroids plus a long-acting beta2 agonist: A randomised double-blind placebo-controlled pivotal phase 2b dose-ranging trial. Lancet.

[76] Castro M., Corren J., Pavord I.D., Maspero J., Wenzel S., Rabe K.F., Busse W.W., Ford L., Sher L., Fitzgerald J.M. (2018). Dupilumab Efficacy and Safety in Moderate-to-Severe Uncontrolled Asthma. New Engl. J. Med..

[77] Pizzichini M.M., Inman M.D., Efthimiadis A., Hargreave F.E., O’Byrne P.M., Nair P., Kjarsgaard M., Pizzichini E. (2009). Mepolizumab for Prednisone-Dependent Asthma with Sputum Eosinophilia. New Engl. J. Med..

[78] Liu A.H., Anderson W.C., Dutmer C.M., Searing D.A., Szefler S.J. (2016). Advances in asthma 2015: Across the lifespan. J. Allergy Clin. Immunol..

[79] FitzGerald J.M., Bleecker E.R., Nair P., Korn S., Ohta K., Lommatzsch M., Ferguson G.T., Busse W.W., Barker P., Sproule S. (2016). Benralizumab, an anti-interleukin-5 receptor a monoclonal antibody, as add-on treatment for patients with severe, uncontrolled, eosinophilic asthma (CALIMA): A randomised, double-blind, placebo-controlled phase 3 trial. Lancet.

[80] Piper E., Brightling C., Niven R., Oh C., Faggioni R., Poon K., She D., Kell C., May R.D., Geba G.P. (2013). A phase II placebo-controlled study of tralokinumab in moderate-to-severe asthma. Eur. Respir. J..

[81] A Hanania N., Noonan M., Corren J., Korenblat P., Zheng Y., Fischer S.K., Cheu M., Putnam W.S., Murray E., Scheerens H. (2015). Lebrikizumab in moderate-to-severe asthma: pooled data from two randomised placebo-controlled studies. Thorax.

[82] Kim M.S., Cho K.-A., Cho Y.J., Woo S.-Y. (2013). Effects of Interleukin-9 Blockade on Chronic Airway Inflammation in Murine Asthma Models. Allergy, Asthma Immunol. Res..

[83] Oh C.K., Leigh R., McLaurin K.K., Kim K., Hultquist M., A Molfino N. (2013). A randomized, controlled trial to evaluate the effect of an anti-interleukin-9 monoclonal antibody in adults with uncontrolled asthma. Respir. Res..

[84] Camargo L.D.N., Righetti R.F., Aristoteles L., Dos Santos T.M., de Souza F.C.R., Fukuzaki S., Cruz M.M., Alonso-Vale M.I.C., Saraiva-Romanholo B.M., Prado C.M. (2017). Effects of Anti-IL-17 on Inflammation, Remodeling, and Oxidative Stress in an Experimental Model of Asthma Exacerbated by LPS. Front. Immunol..

[85] Busse W.W., Holgate S., Kerwin E., Chon Y., Feng J., Lin J., Lin S.L. (2013). Randomized, double-blind, placebo-controlled study of brodalumab, a human anti-IL-17 receptor monoclonal antibody, in moderate to severe asthma. Am. J. Respir. Crit. Care Med..

[86] McMillan S.J., Xanthou G., Lloyd C.M. (2005). Manipulation of allergen-induced airway remodeling by treatment with anti-TGF-beta antibody: effect on the Smad signaling pathway. J. Immunol..

[87] Krug N., Hohlfeld J.M., Kirsten A.-M., Kornmann O., Beeh K.M., Kappeler D., Korn S., Ignatenko S., Timmer W., Rogon C. (2015). Allergen-Induced Asthmatic Responses Modified by a GATA3-Specific DNAzyme. New Engl. J. Med..

[88] Gauvreau G.M., Pageau R., Seguin R., Carballo D., Gauthier J., D’Anjou H., Campbell H., Watson R., Mistry M., Parry-Billings M. (2011). Dose-response effects of TPI ASM8 in asthmatics after allergen. Allergy.

[89] Wu B.-Y., Liu C.-T., Hung Y.-C., Hu W.-L. (2016). Complementary therapy with traditional Chinese medicine for childhood asthma. Asthma—From Childhood Asthma to ACOS Phenotypes.

[90] Liu L., Wang L.P., He S., Ma Y. (2018). Immune Homeostasis: Effects of Chinese Herbal Formulae and Herb-Derived Compounds on Allergic Asthma in Different Experimental Models. Chin. J. Integr. Med..

[91] Srivastava K.D., Dunkin D., Liu C., Yang N., Miller R.L., Sampson H.A., Li X.M. (2014). Effect of Antiasthma Simplified Herbal Medicine Intervention on neutrophil predominant airway inflammation in a ragweed sensitized murine asthma model. Ann. Allergy Asthma Immunol..

[92] Busse P.J., Schofield B., Birmingham N., Yang N., Wen M.-C., Zhang T., Srivastava K., Li X.-M. (2010). The traditional Chinese herbal formula ASHMI inhibits allergic lung inflammation in antigen-sensitized and antigen-challenged aged mice. Ann. Allergy, Asthma Immunol..

[93] Srivastava K., Zhang T., Yang N., Sampson H., Li X.M. (2010). Anti-Asthma Simplified Herbal Medicine Intervention-induced long-lasting tolerance to allergen exposure in an asthma model is interferon-gamma, but not transforming growth factor-beta dependent. Clinical and experimental allergy. J. B. Soc. Allergy Clin. Immunol..

[94] Li X.-M., Huang C.-K., Zhang T.-F., Teper A.A., Srivastava K., Schofield B.H., Sampson H.A. (2000). The Chinese herbal medicine formula MSSM-002 suppresses allergic airway hyperreactivity and modulates TH1/TH2 responses in a murine model of allergic asthma. J. Allergy Clin. Immunol..

[95] Yun L., Xin-sheng F., Jing-hua Y., Li X., Shan-shan W. (2014). CD4+ CD25+ FOXP3+ T cells, Foxp3 gene and protein expression contribute to antiasthmatic effects of San’ao decoction in mice model of asthma. Phytomedicine.

[96] Wei Y., Luo Q.L., Sun J., Chen M.X., Liu F., Dong J.C. (2015). Bu-Shen-Yi-Qi formulae suppress chronic airway inflammation and regulate Th17/Treg imbalance in the murine ovalbumin asthma model. J. Ethnopharmacol..

[97] Lin Z., Jiu-lue H. (2012). Effect of Xiaoqinglong Decoction on Airway Inflammation and IL-4, IFN-γ in the BALF of Mouse Asthmatic Model. Chin. J. Exp. Tradit. Med. Formul..

[98] Kuo Y.-C., Tsai W.-J., Wang J.-Y., Chang S.-C., Lin C.-Y., Shiao M.-S. (2001). Regulation of bronchoalveolar lavage fluids cell function by the immunomodulatory agents from Cordyceps sinensis. Life Sci..

[99] Li L., Hou X., Xu R., Liu C., Tu M. (2017). Research review on the pharmacological effects of astragaloside IV. Fundam. Clin. Pharmacol..

[100] Abe Y., Hashimoto S., Horie T. (1999). Curcumin Inhibition of Inflammatory Cytokine Production by Human Peripheral Blood Monocytes and Alveolar Macrophages. Pharmacol. Res..

[101] Yuan S., Cao S., Jiang R., Liu R., Bai J., Hou Q. (2014). FLLL31, a derivative of curcumin, attenuates airway inflammation in a multi-allergen challenged mouse model. Int. Immunopharmacol..

[102] Kim J.Y., Kim H.J., Kim S.M., Park K.R., Jang H.J., Lee E.H., Jung S.H., Ahn K.S. (2011). Methylene chloride fraction of the leaves of Thuja orientalis inhibits in vitro inflammatory biomarkers by blocking NF-kappaB and p38 MAPK signaling and protects mice from lethal endotoxemia. J. Ethnopharmacol..

[103] Jung H.S., Kim M.H., Gwak N.G., Im Y.S., Lee K.Y., Sohn Y., Choi H., Yang W.M. (2012). Antiallergic effects of Scutellaria baicalensis on inflammation in vivo and in vitro. J. Ethnopharmacol..

[104] Zhao Y.L., Shang J.H., Pu S.B., Wang H.S., Wang B., Liu L., Liu Y.P., Shen H.M., Luo X.D. (2016). Effect of total alkaloids from Alstonia scholaris on airway inflammation in rats. J. Ethnopharmacol..

[105] Shen H.H., Wang K., Li W., Ying Y.H., Gao G.X., Li X.B., Huang H.Q. (2008). Astragalus Membranaceus prevents airway hyperreactivity in mice related to Th2 response inhibition. J. Ethnopharmacol..

[106] Qi Y., Gao F., Hou L., Wan C. (2017). Anti-Inflammatory and Immunostimulatory Activities of Astragalosides. Am. J. Chin. Med..

[107] Huang X., Tang L., Wang F., Song G. (2014). Astragaloside IV attenuates allergic inflammation by regulation Th1/Th2 cytokine and enhancement CD4(+)CD25(+)Foxp3 T cells in ovalbumin-induced asthma. Immunobiology.

[108] Qiu Y.Y., Zhu J.X., Bian T., Gao F., Qian X.F., Du Q., Yuan M.Y., Sun H., Shi L.Z., Yu M.H. (2014). Protective effects of astragaloside IV against ovalbumin-induced lung inflammation are regulated/mediated by T-bet/GATA-3. Pharmacology.

[109] Sung J.E., Lee H.A., Kim J.E., Yun W.B., An B.S., Yang S.Y., Kim D.S., Lee C.Y., Lee H.S., Bae C.J. (2017). Saponin-enriched extract of Asparagus cochinchinensis alleviates airway inflammation and remodeling in ovalbumin-induced asthma model. Int. J. Mol. Med..

[110] Xiong Y.Y., Wu F.H., Wang J.S., Li J., Kong L.Y. (2012). Attenuation of airway hyperreactivity and T helper cell type 2 responses by coumarins from Peucedanum praeruptorum Dunn in a murine model of allergic airway inflammation. J. Ethnopharmacol..

[111] Xiong Y., Wang J., Wu F., Li J., Zhou L., Kong L. (2012). Effects of (+/-)-praeruptorin A on airway inflammation, airway hyperresponsiveness and NF-kappaB signaling pathway in a mouse model of allergic airway disease. Eur. J. Pharmacol..

[112] Xiong Y.Y., Wang J.S., Wu F.H., Li J., Kong L.Y. (2012). The effects of (+/-)-Praeruptorin A on airway inflammation, remodeling and transforming growth factor-beta1/Smad signaling pathway in a murine model of allergic asthma. Int. Immunopharmacol..

[113] Park S., Park M.-S., Jung K.-H., Song J., Kim Y.A., Cho H.J., Min B.-I., Bae H. (2014). Treatment with Pyranopyran-1, 8-Dione Attenuates Airway Responses in Cockroach Allergen Sensitized Asthma in Mice. PLoS ONE.

[114] Yang N., Patil S., Zhuge J., Wen M.-C., Bolleddula J., Doddaga S., Goldfarb J., Sampson H.A., Li X.-M. (2013). Glycyrrhiza uralensis flavonoids present in anti-asthma formula, ASHMI™, inhibit memory Th2 responses in vitro and in vivo. Phytother. Res. PTR.

[115] Wei M., Chu X., Guan M., Yang X., Xie X., Liu F., Chen C., Deng X. (2013). Protocatechuic acid suppresses ovalbumin-induced airway inflammation in a mouse allergic asthma model. Int. Immunopharmacol..

[116] Chu X., Wei M., Yang X., Cao Q., Xie X., Guan M., Wang D., Deng X. (2012). Effects of an anthraquinone derivative from Rheum officinale Baill, emodin, on airway responses in a murine model of asthma. Food Chem. Toxicol..

[117] Lee C.-C., Wang C.-N., Lai Y.-T., Kang J.-J., Liao J.-W., Chiang B.-L., Chen H.-C., Cheng Y.-W. (2010). Shikonin inhibits maturation of bone marrow-derived dendritic cells and suppresses allergic airway inflammation in a murine model of asthma. Br. J. Pharmacol..

[118] Chen M.L., Lin B.F. (2007). Effects of triterpenoid-rich extracts of Ganoderma tsugae on airway hyperreactivity and Th2 responses in vivo. Int. Arch. Allergy Immunol..

[119] Ji N.F., Xie Y.C., Zhang M.S., Zhao X., Cheng H., Wang H., Yin K.S., Huang M. (2014). Ligustrazine corrects Th1/Th2 and Treg/Th17 imbalance in a mouse asthma model. Int. Immunopharmacol..

[120] Do J.S., Hwang J.K., Seo H.J., Woo W.H., Nam S.Y. (2006). Antiasthmatic activity and selective inhibition of type 2 helper T cell response by aqueous extract of semen armeniacae amarum. Immunopharmacol. Immunotoxicol..

[121] Jung H.W., Kang S.Y., Kang J.S., Kim A.R., Woo E.R., Park Y.K. (2014). Effect of Kuwanon G isolated from the root bark of Morus alba on ovalbumin-induced allergic response in a mouse model of asthma. Phytother. Res..

[122] Ok I.S., Kim S.H., Kim B.K., Lee J.C., Lee Y.C. (2009). Pinellia ternata, Citrus reticulata, and their combinational prescription inhibit eosinophil infiltration and airway hyperresponsiveness by suppressing CCR3+ and Th2 cytokines production in the ovalbumin-induced asthma model. Mediat. Inflamm..

[123] Lee M.Y., Shin I.S., Jeon W.Y., Lim H.S., Kim J.H., Ha H. (2013). Pinellia ternata Breitenbach attenuates ovalbumin-induced allergic airway inflammation and mucus secretion in a murine model of asthma. Immunopharmacol. Immunotoxicol..

[124] Lee M.Y., Lee J.A., Seo C.S., Ha H., Lee N.H., Shin H.K. (2011). Protective effects of Mentha haplocalyx ethanol extract (MH) in a mouse model of allergic asthma. Phytother. Res..

[125] Choi J.H., Hwang Y.P., Lee H.S., Jeong H.G. (2009). Inhibitory effect of Platycodi Radix on ovalbumin-induced airway inflammation in a murine model of asthma. Food Chem. Toxicol..

[126] Han E.H., Park J.H., Kim J.Y., Chung Y.C., Jeong H.G. (2009). Inhibitory mechanism of saponins derived from roots of Platycodon grandiflorum on anaphylactic reaction and IgE-mediated allergic response in mast cells. Food Chem. Toxicol..

[127] Jang H.Y., Ahn K.S., Park M.J., Kwon O.K., Lee H.K., Oh S.R. (2012). Skullcapflavone II inhibits ovalbumin-induced airway inflammation in a mouse model of asthma. Int. Immunopharmacol..

[128] Rosa S.I.G., Rios-Santos F., Balogun S.O., de Almeida D.A.T., Damazo A.S., da Cruz T.C.D., Pavan E., Barbosa R.D.S., Alvim T.D.C., Soares I.M. (2017). Hydroethanolic extract from Echinodorus scaber Rataj leaves inhibits inflammation in ovalbumin-induced allergic asthma. J. Ethnopharmacol..

[129] Jung W.K., Lee D.Y., Choi Y.H., Yea S.S., Choi I., Park S.G., Seo S.K., Lee S.W., Lee C.M., Kim S.K. (2008). Caffeic acid phenethyl ester attenuates allergic airway inflammation and hyperresponsiveness in murine model of ovalbumin-induced asthma. Life Sci..

[130] Chen C.G., Wang H.Y., Dai Y., Wang J.L., Xu W.H. (2013). Tripterygium polyglycosid attenuates the established airway inflammation in asthmatic mice. Chin. J. Integr. Med..

[131] Liu Q. (2011). Triptolide and its expanding multiple pharmacological functions. Int. Immunopharmacol..

[132] Chen M., Lv Z., Zhang W., Huang L., Lin X., Shi J., Zhang W., Liang R., Jiang S. (2015). Triptolide suppresses airway goblet cell hyperplasia and Muc5ac expression via NF-kappaB in a murine model of asthma. Mol. Immunol..

[133] Sy L.B., Wu Y.L., Chiang B.L., Wang Y.H., Wu W.M. (2006). Propolis extracts exhibit an immunoregulatory activity in an OVA-sensitized airway inflammatory animal model. Int. Immunopharmacol..

[134] Hsieh C.C., Hsiao H.B., Lin W.C. (2010). A standardized aqueous extract of Anoectochilus formosanus modulated airway hyperresponsiveness in an OVA-inhaled murine model. Phytomedicine.

[135] Huang W.C., Kuo M.L., Li M.L., Yang R.C., Liou C.J., Shen J.J. (2008). Gynostemma pentaphyllum decreases allergic reactions in a murine asthmatic model. Am. J. Chin. Med..

[136] Du Q., Gu X.Y., Feng G.Z., Shen L., Cui J., Cai J.K., Huang M., Yin K.S. (2011). Effects of astragaloside IV on the expressions of transforming growth factor-beta1 and thymic stromal lymphopoietin in a murine model of asthma. Zhonghua Yi Xue Za Zhi.

[137] Tanaka T., Takahashi R. (2013). Flavonoids and asthma. Nutrients.

[138] Karunaweera N., Raju R., Gyengesi E., Munch G. (2015). Plant polyphenols as inhibitors of NF-kappaB induced cytokine production-a potential anti-inflammatory treatment for Alzheimer’s disease?. Front. Mol. Neurosci..

[139] Marzulli G., Magrone T., Kawaguchi K., Kumazawa Y., Jirillo E. (2012). Fermented grape marc (FGM): Immunomodulating properties and its potential exploitation in the treatment of neurodegenerative diseases. Curr. Pharm. Des..

[140] Gong J.H., Shin D., Han S.Y., Kim J.L., Kang Y.H. (2012). Kaempferol suppresses eosionphil infiltration and airway inflammation in airway epithelial cells and in mice with allergic asthma. J. Nutr..

[141] Heras B., Hortelano S. (2009). Molecular Basis of the Anti-Inflammatory Effects of Terpenoids. Inflamm. Allergy-Drug Targets.

[142] Bae Y., Lee S., Kim S.H. (2011). Chrysin suppresses mast cell-mediated allergic inflammation: Involvement of calcium, caspase-1 and nuclear factor-kappaB. Toxicol. Appl. Pharmacol..

[143] Medeiros K.C.P., Faustino L., Borduchi E., Nascimento R.J., Silva T.M.S., Gomes E., Piuvezam M.R., Russo M. (2009). Preventive and curative glycoside kaempferol treatments attenuate the TH2-driven allergic airway disease. Int. Immunopharmacol..

[144] Higa S., Hirano T., Kotani M., Matsumoto M., Fujita A., Suemura M., Kawase I., Tanaka T. (2003). Fisetin, a flavonol, inhibits TH2-type cytokine production by activated human basophils. J. Allergy Clin. Immunol..

[145] Huang W., Li M.L., Xia M.Y., Shao J.Y. (2018). Fisetin-treatment alleviates airway inflammation through inhbition of MyD88/NF-kappaB signaling pathway. Int. J. Mol. Med..

[146] Leyva-Lopez N., Gutierrez-Grijalva E.P., Ambriz-Perez D.L., Heredia J.B. (2016). Flavonoids as Cytokine Modulators: A Possible Therapy for Inflammation-Related Diseases. Int. J. Mol. Sci..

[147] Kempuraj D., Madhappan B., Christodoulou S., Boucher W., Cao J., Papadopoulou N., Cetrulo C.L., Theoharides T.C. (2005). Flavonols inhibit proinflammatory mediator release, intracellular calcium ion levels and protein kinase C theta phosphorylation in human mast cells. Br. J. Pharmacol..

[148] Segawa S., Yasui K., Takata Y., Kurihara T., Kaneda H., Watari J. (2006). Flavonoid glycosides extracted from hop (Humulus lupulus L.) as inhibitors of chemical mediator release from human basophilic KU812 cells. Biosci. Biotechnol. Biochem..

[149] Kandhare A.D., Liu Z., Mukherjee A.A., Bodhankar S.L. (2019). Therapeutic potential of Morin in Ovalbumin-induced allergic asthma via modulation of SUMF2/IL-13 and BLT2/NF-kB signaling pathway. Curr. Mol. Pharmacol..

[150] Liu C., Zhu L., Fukuda K., Ouyang S., Chen X., Wang C., Zhang C.J., Martin B., Gu C., Qin L. (2017). The flavonoid cyanidin blocks binding of the cytokine interleukin-17A to the IL-17RA subunit to alleviate inflammation in vivo. Sci Signal.

[151] Liu L.L., Li F.H., Zhang Y., Zhang X.F., Yang J. (2017). Tangeretin has anti-asthmatic effects via regulating PI3K and Notch signaling and modulating Th1/Th2/Th17 cytokine balance in neonatal asthmatic mice. Braz. J. Med. Biol. Res..

[152] Liu L.L., Zhang Y., Zhang X.F., Li F.H. (2018). Influence of rutin on the effects of neonatal cigarette smoke exposure-induced exacerbated MMP-9 expression, Th17 cytokines and NF-kappaB/iNOS-mediated inflammatory responses in asthmatic mice model. Korean J. Physiol. Pharmacol..

[153] Xu L., Li J., Zhang Y., Zhao P., Zhang X. (2017). Regulatory effect of baicalin on the imbalance of Th17/Treg responses in mice with allergic asthma. J. Ethnopharmacol..

[154] Shi Y., Dai J., Liu H., Li R.R., Sun P.L., Du Q., Pang L.L., Chen Z., Yin K.S. (2009). Naringenin inhibits allergen-induced airway inflammation and airway responsiveness and inhibits NF-kappaB activity in a murine model of asthma. Can. J. Physiol. Pharmacol..

[155] Ci X., Zhong W., Ren H., Wen Z., Li D., Peng L. (2015). Esculentoside A Attenuates Allergic Airway Inflammation via Activation of the Nrf-2 Pathway. Int. Arch. Allergy Immunol..

[156] Hamalainen M., Nieminen R., Vuorela P., Heinonen M., Moilanen E. (2007). Anti-inflammatory effects of flavonoids: Genistein, kaempferol, quercetin, and daidzein inhibit STAT-1 and NF-kappaB activations, whereas flavone, isorhamnetin, naringenin, and pelargonidin inhibit only NF-kappaB activation along with their inhibitory effect on iNOS expression and NO production in activated macrophages. Mediat. Inflamm..

[157] Lee J.S., Lee C.M., Jeong Y.I., Jung I.D., Kim B.H., Seong E.Y., Kim J.I., Choi I.W., Chung H.Y., Park Y.M. (2007). D-pinitol regulates Th1/Th2 balance via suppressing Th2 immune response in ovalbumin-induced asthma. FEBS Lett..

[158] Abdelaziz R.R., Elmahdy M.K., Suddek G.M. (2018). Flavocoxid attenuates airway inflammation in ovalbumin-induced mouse asthma model. Chem. Biol. Interact..

[159] Li J., Zhang B. (2013). Apigenin protects ovalbumin-induced asthma through the regulation of Th17 cells. Fitoterapia.

[160] Jin M., Yang J.H., Lee E., Lu Y., Kwon S., Son K.H., Son J.K., Chang H.W. (2009). Antiasthmatic activity of luteolin-7-O-glucoside from Ailanthus altissima through the downregulation of T helper 2 cytokine expression and inhibition of prostaglandin E2 production in an ovalbumin-induced asthma model. Biol. Pharm. Bull..

[161] Ebrahimi H., Fallahi M., Khamaneh A.M., Ebrahimi Saadatlou M.A., Saadat S., Keyhanmanesh R. (2016). Effect of alpha-Hederin on IL-2 and IL-17 mRNA and miRNA-133a Levels in Lungs of Ovalbumin-Sensitized Male Rats. Drug Dev. Res..

[162] Fallahi M., Keyhanmanesh R., Khamaneh A.M., Saadatlou M.A.E., Saadat S., Ebrahimi H. (2016). Effect of Alpha-Hederin, the active constituent of Nigella sativa, on miRNA-126, IL-13 mRNA levels and inflammation of lungs in ovalbumin-sensitized male rats. Avicenna J. Phytomed..

[163] Junchao Y., Zhen W., Yuan W., Liying X., Libin J., Yuanhong Z., Wei Z., Ruilin C., Lu Z. (2017). Anti- trachea inflammatory effects of diosgenin from Dioscorea nipponica through interactions with glucocorticoid receptor alpha. J. Int. Med. Res..

[164] Ninave P.B., Patil S.D. (2019). Antiasthmatic potential of Zizyphus jujuba Mill and Jujuboside B. - Possible role in the treatment of asthma. Respir. Physiol. Neurobiol..

[165] Liu C., Yang N., Song Y., Wang L., Zi J., Zhang S., Dunkin D., Busse P., Weir D., Tversky J. (2015). Ganoderic acid C1 isolated from the anti-asthma formula, ASHMI suppresses TNF-alpha production by mouse macrophages and peripheral blood mononuclear cells from asthma patients. Int. Immunopharmacol..

[166] Fernandez M.A., de las Heras B., Garcia M.D., Saenz M.T., Villar A. (2001). New insights into the mechanism of action of the anti-inflammatory triterpene lupeol. J. Pharm. Pharmacol..

[167] Vasconcelos J.F., Teixeira M.M., Barbosa-Filho J.M., Lucio A.S., Almeida J.R., de Queiroz L.P., Ribeiro-Dos-Santos R., Soares M.B. (2008). The triterpenoid lupeol attenuates allergic airway inflammation in a murine model. Int. Immunopharmacol..

[168] Liu Z., Liu X., Sang L., Liu H., Xu Q., Liu Z. (2015). Boswellic acid attenuates asthma phenotypes by downregulation of GATA3 via pSTAT6 inhibition in a murine model of asthma. Int. J. Clin. Exp. Pathol..

[169] Jung H.W., Chung Y.S., Kim Y.S., Park Y.K. (2007). Celastrol inhibits production of nitric oxide and proinflammatory cytokines through MAPK signal transduction and NF-kappaB in LPS-stimulated BV-2 microglial cells. Exp.Mol. Med..

[170] Lindner I., Meier C., Url A., Unger H., Grassauer A., Prieschl-Grassauer E., Doerfler P. (2010). Beta-escin has potent anti-allergic efficacy and reduces allergic airway inflammation. BMC Immunol..

[171] Isik S., Karaman M., Micili S.C., Caglayan-Sozmen S., Bagriyanik H.A., Arikan-Ayyildiz Z., Uzuner N., Karaman O. (2018). Sinomenine ameliorates the airway remodelling, apoptosis of airway epithelial cells, and Th2 immune response in a murine model of chronic asthma. Allergol. Immunopathol..

[172] Kim S.H., Hong J.H., Lee Y.C. (2015). Chelidonine, a principal isoquinoline alkaloid of Chelidonium majus, attenuates eosinophilic airway inflammation by suppressing IL-4 and eotaxin-2 expression in asthmatic mice. Pharmacol. Rep..

[173] Song Y., Wu Y., Li X., Shen Y., Ding Y., Zhu H., Liu F., Yu K., Sun L., Qian F. (2018). Protostemonine attenuates alternatively activated macrophage and DRA-induced asthmatic inflammation. Biochem. Pharmacol..

[174] Xiong L., Fang Z.Y., Tao X.N., Bai M., Feng G. (2007). Effect and mechanism of ligustrazine on Th1/Th2 cytokines in a rat asthma model. Am. J. Chin. Med..

[175] Gibbs B.F. (2009). Differential modulation of IgE-dependent activation of human basophils by ambroxol and related secretolytic analogues. Int. J. Immunopathol. Pharmacol..

[176] Li Z., Zheng J., Zhang N., Li C. (2016). Berberine improves airway inflammation and inhibits NF-kappaB signaling pathway in an ovalbumin-induced rat model of asthma. J. Asthma.

[177] Kim S.H., Park H.J., Lee C.M., Choi I.W., Moon D.O., Roh H.J., Lee H.K., Park Y.M. (2006). Epigallocatechin-3-gallate protects toluene diisocyanate-induced airway inflammation in a murine model of asthma. FEBS Lett..

[178] Rogerio A.P., Fontanari C., Borducchi E., Keller A.C., Russo M., Soares E.G., Albuquerque D.A., Faccioli L.H. (2008). Anti-inflammatory effects of Lafoensia pacari and ellagic acid in a murine model of asthma. Eur. J. Pharmacol..

[179] Zhou E., Fu Y., Wei Z., Yang Z. (2014). Inhibition of allergic airway inflammation through the blockage of NF-kappaB activation by ellagic acid in an ovalbumin-induced mouse asthma model. Food Funct..

[180] Lee M., Kim S., Kwon O.K., Oh S.R., Lee H.K., Ahn K. (2009). Anti-inflammatory and anti-asthmatic effects of resveratrol, a polyphenolic stilbene, in a mouse model of allergic asthma. Int. Immunopharmacol..

[181] Kim S.Y., Moon K.A., Jo H.Y., Jeong S., Seon S.H., Jung E., Cho Y.S., Chun E., Lee K.Y. (2012). Anti-inflammatory effects of apocynin, an inhibitor of NADPH oxidase, in airway inflammation. Immunol. Cell Biol..

[182] Chen M., Lv Z., Jiang S. (2011). The effects of triptolide on airway remodelling and transforming growth factor-beta(1)/Smad signalling pathway in ovalbumin-sensitized mice. Immunology.

[183] Bao Z., Guan S., Cheng C., Wu S., Wong S.H., Kemeny D.M., Leung B.P., Wong W.S. (2009). A novel antiinflammatory role for andrographolide in asthma via inhibition of the nuclear factor-kappaB pathway. Am. J. Respir. Crit. Care Med..

[184] Munroe M.E., Businga T.R., Kline J.N., Bishop G.A. (2010). Anti-inflammatory effects of the neurotransmitter agonist Honokiol in a mouse model of allergic asthma. J. Immunol..

[185] Keyhanmanesh R., Boskabady M.H., Khamneh S., Doostar Y. (2010). Effect of thymoquinone on the lung pathology and cytokine levels of ovalbumin-sensitized guinea pigs. Pharmacol. Rep..

[186] Li J., Zhang F., Li J. (2015). The Immunoregulatory Effects of Traditional Chinese Medicine on Treatment of Asthma or Asthmatic Inflammation. Am. J. Chin. Med..

[187] Demlova R., Valik D., Obermannova R., ZdraZilova-Dubska L. (2016). The safety of therapeutic monoclonal antibodies: Implications for cancer therapy including immuno-checkpoint inhibitors. Physiol. Res..

[188] Byard R.W., Musgrave I., Maker G., Bunce M. (2017). What risks do herbal products pose to the Australian community?. Med. J. Aust..

